# Comparative metabolic profiling of the mycelium and fermentation broth of *Penicillium restrictum* from *Peucedanum praeruptorum* rhizosphere

**DOI:** 10.1111/1758-2229.13286

**Published:** 2024-06-06

**Authors:** Yuanyuan Wang, Ranran Liao, Haoyu Pan, Xuejun Wang, Xiaoting Wan, Bangxing Han, Cheng Song

**Affiliations:** ^1^ School of Pharmacy Anhui University of Chinese Medicine Hefei China; ^2^ Anhui Dabieshan Academy of Traditional Chinese Medicine, Anhui Engineering Research Center for Eco‐agriculture of Traditional Chinese Medicine, College of Biological and Pharmaceutical Engineering West Anhui University Luan China; ^3^ School of Life Science Anhui Agricultural University Hefei China

## Abstract

Microorganisms in the rhizosphere, particularly arbuscular mycorrhiza, have a broad symbiotic relationship with their host plants. One of the major fungi isolated from the rhizosphere of *Peucedanum praeruptorum* is *Penicillium restrictum*. The relationship between the metabolites of *P. restrictum* and the root exudates of *P. praeruptorum* is being investigated. The accumulation of metabolites in the mycelium and fermentation broth of *P. restrictum* was analysed over different fermentation periods. Non‐targeted metabolomics was used to compare the differences in intracellular and extracellular metabolites over six periods. There were significant differences in the content and types of mycelial metabolites during the incubation. Marmesin, an important intermediate in the biosynthesis of coumarins, was found in the highest amount on the fourth day of incubation. The differential metabolites were screened to obtain 799 intracellular and 468 extracellular differential metabolites. Kyoto Encyclopedia of Genes and Genomes pathway enrichment analysis showed that the highly enriched extracellular metabolic pathways were alanine, aspartate and glutamate metabolism, glyoxylate and dicarboxylate metabolism, and terpenoid backbone biosynthesis. In addition, the enrichment analysis associated with intracellular and extracellular ATP‐binding cassette transporter proteins revealed that some ATP‐binding cassette transporters may be involved in the transportation of certain amino acids and carbohydrates. Our results provide some theoretical basis for the regulatory mechanisms between the rhizosphere and the host plant and pave the way for the heterologous production of furanocoumarin.

## Graphical abstract

*Penicillium restrictum* is a fungus isolated from the rhizosphere of *Peucedanum praeruptorum*. Non‐targeted metabolomics revealed the main components in the mycelium and fermentation broth of *P. restrictum*, especially the accumulation pattern of coumarins.

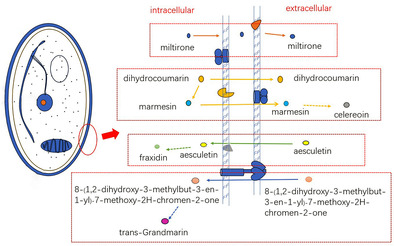

## INTRODUCTION

Rhizosphere microbial communities play an important and direct role in soil health and plant growth (Kong & Liu, 2022; Li et al., 2019; Liu et al., 2020). Fungi are an important part of the soil microbiome and are involved in biochemical cycles and the decomposition of complex organic matter (Mayer et al., 2021). Compared to bacterial communities, fungal communities are more representative and important for plant growth in disturbed soil environments (Zheng et al., 2019). Previous studies showed that the fungal community was the most important predictor of plant health during monoculture, as it could drive more complex, healthy plant‐related networks (Ding et al., 2023). Prior to studying the processes of rhizosphere fungal and plant interactions, it is essential to identify the secondary metabolites generated by rhizosphere fungi. Given the intricate character of the secondary metabolites produced by the rhizosphere fungus, advanced histological approaches can be employed to investigate the growth alterations of endophytes.

Metabolomics focuses on endogenous small‐molecule metabolites in organisms based on a combination of high‐throughput analysis and chemometrics (Paris et al., 2018). This technique is commonly used in research fields such as gene function analysis, metabolic pathways and regulatory mechanisms. It employs many analytical instruments to examine a wide range of metabolites in a specific biological sample (Pinu, 2016). The metabolome represents the most direct reflection of an organism's phenotype, lying below the transcriptome and proteome. The metabolome provides essential insights into the interactions between plants and rhizosphere microbiota, including the impact of rhizosphere fungal inoculation on the plant metabolome (Mishra et al., 2022). Metabolomics has been successfully used as an important tool for the analysis of complex secondary metabolites in microorganisms. Metabolomics has been successfully used to build metabolite fingerprints by comparing solid and liquid culture extracts of the endophyte *Curvularia* sp. (Tawfike et al., 2017). Metabolomics can also be used to unravel how the grape endophyte *Alternaria* sp. MG1 mediates phenylpropanoid biosynthesis in response to starvation (Lu et al., 2019). Metabolomics can also be combined with genomics, thus allowing for the localization of key genes. Based on 16S rRNA sequencing and the phylogeny of seven *Bacillus* strains isolated from *Calendula officinalis*, the result showed that *Bacillus halotolerans* Cal.l.30 has a large group of genes involved in secondary metabolism biosynthesis. These genes include a lot of CAZyme genes that are active against fungi and can synthesize some valuable metabolites that are antimicrobial (Tsalgatidou et al., 2022). The application of metabolomics for the analysis of secondary metabolites is now well‐established and efficient.

The traditional Chinese medicine ‘QianHu’ is the dried root of *Peucedanum praeruptorum* Dunn from the Apiaceae family. It has long been used to treat respiratory disorders such as cough, phlegm, and respiratory accumulation (Ishii et al., 2008). Currently, numerous studies are concentrating on its chemical composition, pharmacology, and pharmacodynamics, as for the isolation and identification of endophytic bacteria, only two new strains have been found in *P. praeruptorum*: *Streptomyces akebiae* sp. nov. and *Mumia xiangluensis* sp. nov. (Mo et al., 2022; Zhou et al., 2016). *Streptomyces akebiae* nov. has grey aerial mycelium and yellow basidiomycelium, and the aerial mycelium has developed a straight to curved spore chain appearance with a smooth surface. *Mumia xiangluensis* sp. nov. is aerobic, non‐motile, Gram‐stain‐positive, and formed from irregular spheroids without spores. A strain belonging to Didymella was previously isolated from traditional culture and was found to produce praeruptorin A, praeruptorin B and praeruptorin E (Liu et al., 2022; Song et al., 2021; Song et al., 2022). Meanwhile, metagenomic sequencing showed a relatively high abundance of *Penicillium restrictum* in the genus *Penicillium*. After fermentation, the fungus was found to produce coumarin‐like compounds, the medicinal components of *P. praeruptorum*. Although the biosynthesis of coumarins has been tentatively demonstrated, the biogenic pathway of furanocoumarins is more challenging to elucidate. Several enzymes that were important for the synthesis of furanocoumarins: prenyltransferase, psoralen synthase and marmesin synthase, did not show any activity when expressed in *Escherichia coli* (Rodrigues et al., 2022; Song et al., 2024).

Rhizosphere microorganisms usually affect the growth and development of plants and the accumulation of secondary metabolites; however, only a few reports exist on the isolation and identification of rhizosphere microorganisms in *P. praeruptorum* and their interacting mechanisms with the host. Using a metabolomics approach, this study systematically investigated the distribution and accumulation of secondary metabolites in *P. restrictum*. *Penicillium restrictum* contained both furancoumarins and pyranocoumarins. Marmesin is the most abundant coumarin component, which acts as an important precursor for the synthesis of most coumarins and has a greater influence on the synthesis of other coumarins, and the highest content is approaching the 4th day of culture, which can be referred to as the best phase for *P. restrictum* inoculation. KEGG pathway analysis showed that a large number of genes involved in secondary metabolite synthesis were significantly enriched. ABC transporter subfamilies may be involved in the transport of amino acids and carbohydrates. *Penicillium restrictum* produces a wide range of coumarins with different structures, which may provide an alternative way for the heterologous production of coumarin and its precursors.

## MATERIALS AND METHODS

### *Screening and culture of* Penicillium restrictum

The endophyte of *P. restrictum* was isolated from the roots of unbolting *P. praeruptorum*. The internally transcribed spacer‐based amplicon sequencing was carried out in accordance with the DNA extraction and sequencing steps. The obtained sequences were spliced and filtered by quality control to obtain effective data (Canarini et al., 2021). Usearch was used to conduct the removal of the non‐amplified region sequence, correction of the error, and division of the sequences into different operational taxonomic units (OTUs) based on their similarity. Statistical analysis of biological information was performed on OTUs at 97% similar levels (Li et al., 2021). Statistical analysis of biological information was performed on OTUs at 97% similar levels (Li et al., 2021). The fungal sequences were classified using ribosomal database project, Silva and NCBI databases. The two sequences have homology, with a score of 99.82%. The accession number of *P. restrictum* is AF033459.1. The isolated *P. restrictum* was cultured with potato glucose agar potato dextrose agar (PDA) medium and potato dextrose broth (PDB) medium, respectively. PDB medium formula: 200 g/L potato and 20 g/L glucose. PDA medium was added with 15–20 g/L of agar in PDB medium.

### *Fermentation and microscopic observation of* P. restrictum

The strains were first incubated in a PDA medium at 28°C without light for 7 days (Martínez‐Salgado et al., 2021). After growing to the full size of the Petri dishes, the endophytic fungal clusters were picked and incubated in conical flasks containing PDB medium at 28°C, cultured for 12 days with the maximum fermentation incubation period (Khamkong et al., 2022). A total of 40 bottles were fermented, and six bottles were sampled every 2 days. Subsequently, the fermentation broth was taken to observe the morphology. The mycelium size, number and fresh and dry weight were measured. The fermentation broth was filtered with four layers of gauze. The filtered fermentation broth was centrifuged, and the precipitate was discarded. The supernatant was mixed and quickly quenched in liquid nitrogen for 30 s, and then put into the −80°C refrigerator for freezing. The filtered mycelium was collected and rinsed with ultrapure water in a cloth funnel. The mycelium was pump‐filtered for 20 min and then weighed after being completely drained. The filtered mycelium was collected into a vacuum tube, freeze‐dried for 3 days and finally weighed as dry weight.

### Metabolomics analysis

The intracellular and extracellular metabolites of *P. restrictum* were investigated and compared over six fermentation periods. A total of 60 samples, including 30 mycelium samples and 30 fermentation broth samples, were selected for metabolomics analysis. The periods, days 2, 4, 6, 8, 10 and 12, were consistent with days after cultures 2, 4, 6, 8, 10 and 12, respectively. Every 100 μL of the samples was transferred to centrifuge tubes. After the addition of 300 μL of extract solution (internal standard mixture), the samples were vortexed for 30 s, sonicated for 10 min in an ice‐water bath and incubated for 1 h at −40°C to precipitate proteins. Then the sample was centrifuged at 13,800 *g* for 15 min at 4°C. The supernatant was transferred to a fresh glass vial for later analysis. The quality control sample was prepared by mixing an equal aliquot of the supernatants from all of the samples. LC–MS/MS analyses were performed using a UHPLC system (Vanquish, Thermo Fisher Scientific) with a UPLC HSS T3 column (2.1 mm × 100 mm, 1.8 μm) coupled to an Orbitrap Exploris 120 mass spectrometer (Orbitrap MS, Thermo). The mobile phase consisted of 5 mmol/L ammonium acetate and 5 mmol/L acetic acid, both in water (A) and acetonitrile (B). The sampler was set at 4°C, and the injection volume was 2 μL. The mass spectrometer was used to acquire MS/MS spectra in information dependent aquisition mode under the control of the acquisition software (Xcalibur, Thermo Fisher Scientific). The acquisition software continuously evaluates the full‐scan MS spectrum. The ESI source conditions were set as follows: sheath gas flow rate as 50 Arb, auxiliary gas flow rate as 15 Arb, capillary temperature 320°C, full MS resolution as 60,000, MS/MS resolution as 15,000, collision energy as 10/30/60 in NCE mode, spray voltage as 3.8 kV (positive) or −3.4 kV (negative), respectively.

### Mass spectrometry data processing and screening of differential compounds

The original data were changed to the mzXML format using ProteoWizard. They were then processed with a custom programme built with R and based on the XCMS database (https://github.com/sneumann/xcms) to find peaks, extract them, align them and combine them. Then it was matched with BiotreeDB (v. 2.1) (Biotree Ltd., Shanghai) for substance annotation, and the cutoff value for algorithmic scoring was set to 0.3. The univariate analyses were performed based on the mass spectral results, including the Student's *t*‐test and fold change (FC) for analysing significant differences between groups. The multivariate analyses were performed with principal components (PCA) and partial least squares discriminant analysis (OPLS‐DA). The significance of the variables in the projected (VIP) values from the OPLS‐DA model was combined with the p‐values and fold change (FC) values analysed by the Student's *t*‐test to confirm the significance of the differential metabolites. Differential compounds were screened according to the following conditions: (1) high contribution to sample classification in partial least squares (VIP >1.25); (2) the difference multiples between groups, that is, the differential genes with FC values >2 and FC values <0.5, changed greatly; (3) the difference between the two groups was statistically significant, that is, p < 0.01. The raw data has been uploaded to Figshare (https://doi.org/10.6084/m9.figshare.24471793.v1) (Wang, 2023).

### KEGG pathway enrichment analysis

The Kyoto Encyclopedia of Genes and Genomes (KEGG) Pathway database (http://www.kegg.jp/kegg/pathway.html) was used for metabolic network enrichment analysis. For functional annotation, the BlastKOALA tool on the KEGG homepage was used for KO annotation and KEGG mapping. After submitting the compound information, *Penicillium rubens* was selected as the reference species. All the pathways were mapped by differential metabolites of the reference species. According to the enrichment of differential metabolites in KEGG pathways, the rich factor (the ratio of the number of differential metabolites annotated in a pathway to the number of all the metabolites in the pathway) was calculated. The larger the value indicated, the larger the enrichment degree focused. A network‐based enrichment analysis was constructed, which included metabolic pathways, modules, enzymes, reactions, and metabolites. In some situations, the targeting of possible enzymes and metabolites can be shown by the intersections of metabolic pathways. After obtaining matching information for each set of contrasting differential metabolites, pathway searches and regulatory interactions network analyses were performed on the KEGG database of the reference species.

## RESULTS

### *Morphology and biomass detection of* Penicillium restrictum

**FIGURE 1:**
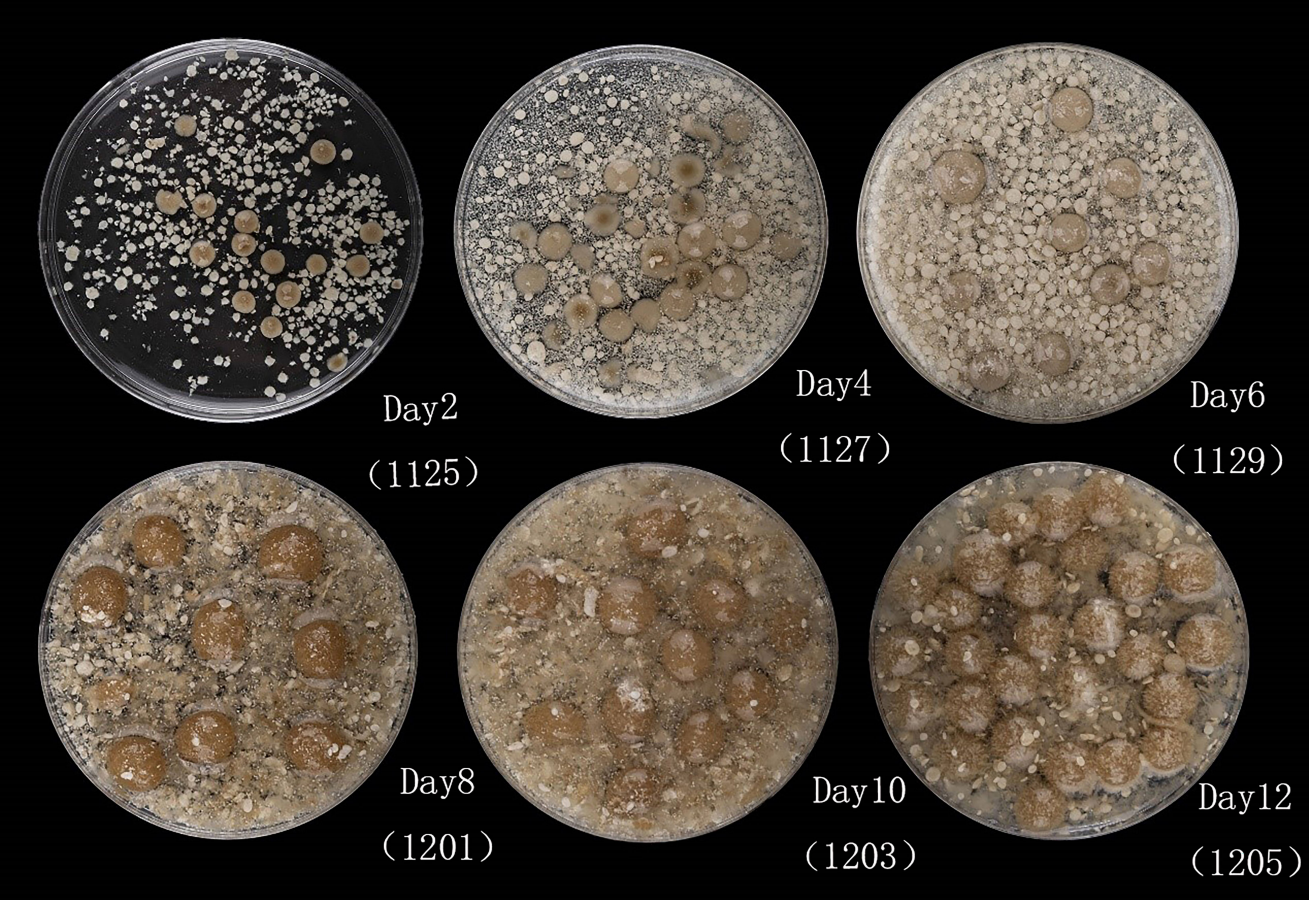
Morphological observation of mycelium after fermentation. The samples were taken at the 2nd, 4th, 6th, 8th, 10th and 12th after fermentation, respectively. The spherical part is a mycelium ball wrapped by hyphae, and the colour of the middle part gradually deepened with the increase of incubation time.

**FIGURE 2:**
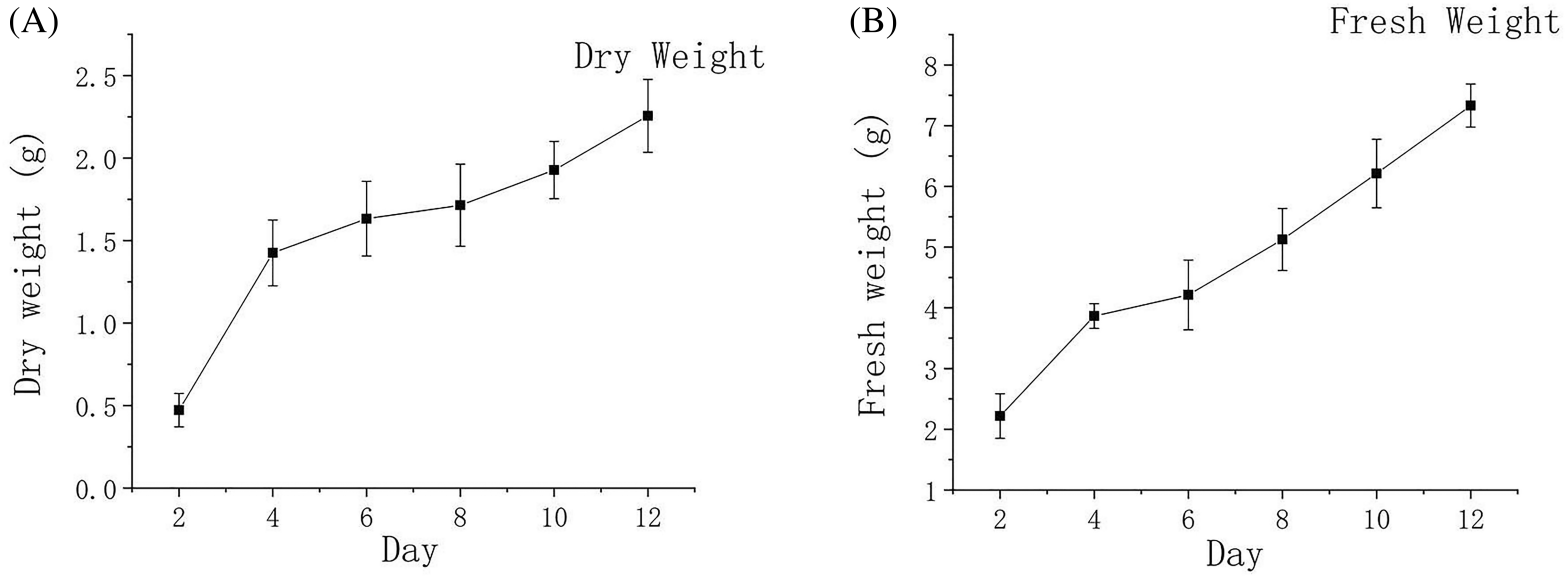
Growth determination of mycelium. (A) Dry weight of mycelium; (B) Fresh weight of mycelium. Each sample was averaged after five biological replicates.

*Penicillium restrictum* was gradually entangled into balls with shaking during the growth process when forming smaller mycelia balls in the early stage. As more and more mycelium was entangled, its morphology became larger. The cell contents increased, the fermentation colour gradually deepened and browned, and the formed mycelium expanded continuously (Figure 1). By measuring the biomass of the shaken flask during incubation from days 2 to 12, the dry and fresh weights of mycelium increased gradually. On the 12th day of culture, both fresh and dry weights reached maximum values of about 7.3 and 2.26 g/dw, respectively (Figure 2A,B). After 12 days of culture, the mycelium continued to consume nutrients, and the weight continued to increase until it was deprived of nutrients, and the rate of weight increase gradually slowed down to no longer increasing.

### PCA and OPLS‐DA analyses of different groups

**FIGURE 3:**
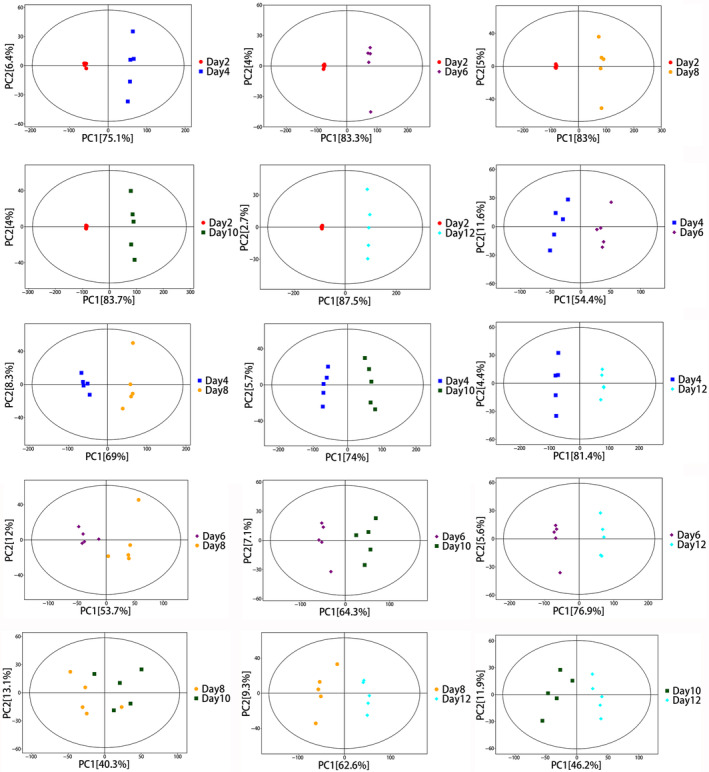
PCA score plots at different times in the fermentation broth.

**FIGURE 4:**
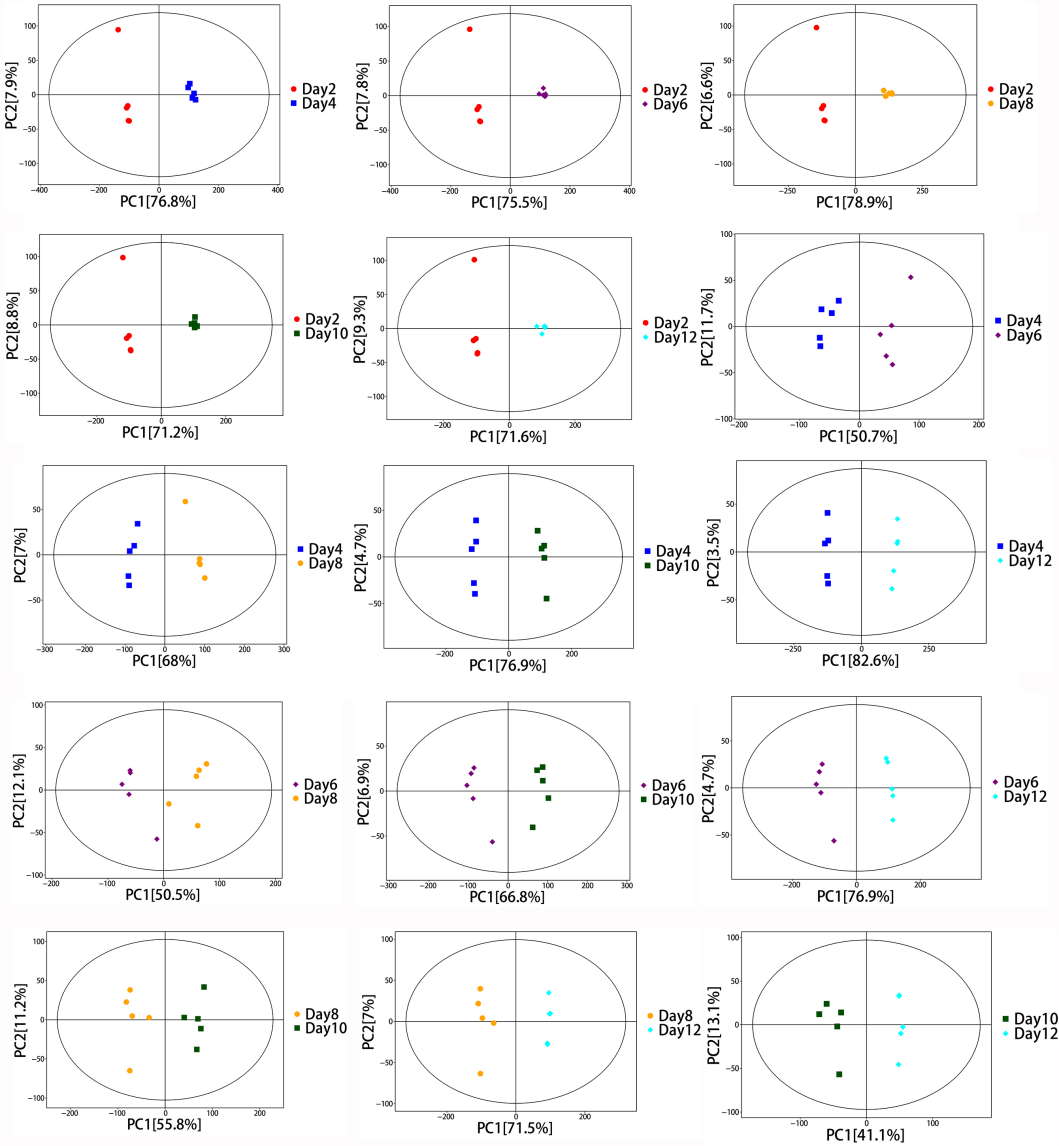
PCA score plots at different times in the mycelium.

**FIGURE 5:**
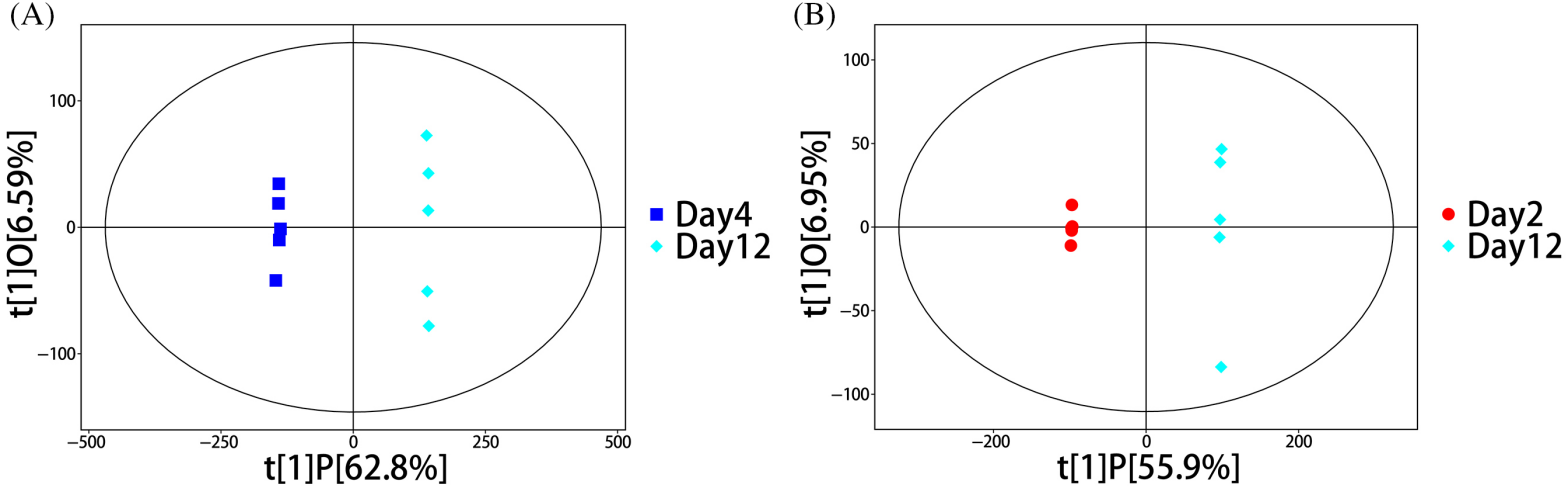
OPLS‐DA score plots of fermentation broth and mycelium.

PCA is an unsupervised multivariate statistical analysis that captures the overall differences and degree of variability among samples. The PCA score indicated that all samples were within the 95% confidence interval (Figures 3 and 4). The horizontal coordinate PC[1] and vertical coordinate PC[2] in the PCA plot indicate the scores of the first and the second principal components respectively. Each scatter represents a sample, and the colours and shape of the scatters denote different groupings, and the closer the sample points are distributed, the indicate the more similar the types and contents of metabolites in the samples. The PCA analysis of the intra‐ and extracellular samples in mycelium and fermentation broth suggested that the intra‐ and extracellular metabolic profiles of the samples tended to have a great trend of separation, which indicated a good degree of separation and reproducibility within the groups. There was a significant difference in the content and type of mycelium metabolites in the process of cultivation. The horizontal coordinate t[1]P in the OPLS‐DA plot indicates the predicted principal component score of the first principal component, demonstrating the differences between sample groups, and the longer distance indicates the big difference. The vertical coordinate t[1]O indicates the orthogonal principal component scores, demonstrating the differences within the sample groups and the closer distance indicates the small difference within the groups and good reproducibility. The separation and reproducibility of samples within groups were good (Figure 5). It was demonstrated that there were significant changes in intracellular and extracellular metabolites of *P. restrictum* at different times.

### *Screening and identification of differential metabolites in* P. restrictum

**FIGURE 6:**
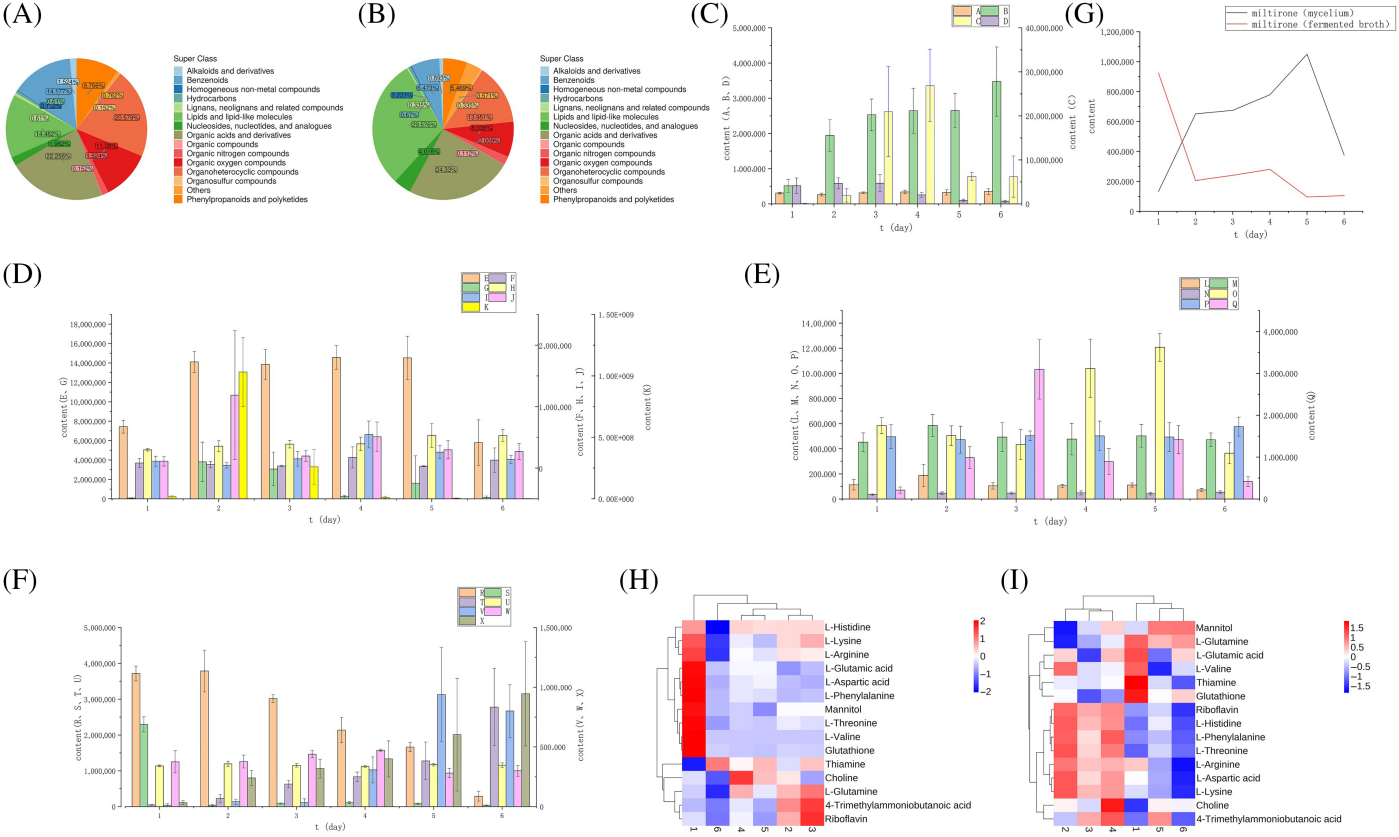
Identification and classification of major differential compounds in fermentation broth and mycelium. (A) Composition and classification of fermentation broth components; (B) Composition and classification of mycelium components; (C) The contents of pyranocoumarins in different periods. A: 8‐(1,2‐dihydroxy‐3‐methylbut‐3‐en‐1‐yl)‐7‐methoxy‐2H‐chromen‐2‐one (fermented broth); B: khellactone (fermented broth), C: *trans*‐Grandmarin (mycelium) D: graveolone (mycelium); (D) The contents of furanocoumarins in different periods. E: 4‐Hydroxy‐8‐methoxy‐2H‐furo[2,3‐h]‐1‐benzopyran‐2‐one (fermented broth); F: aflatoxin P1(fermented broth); G: celereoin (fermented broth); H: mukurozidiol (fermented broth); (E) I: 8‐geranyloxypsoralen (mycelium); J: archangelin (mycelium); K: marmesin (mycelium); (E) The contents of simple coumarins different periods. L: 3‐*O*‐Acetylepisamarcandin(fermented broth); M: 5‐hydroxy‐6‐methoxycellulose(fermented broth); H: mukurozidiol(fermented broth); H: mukurozidiol (fermented broth); (E) Hydroxy‐6‐methoxycoumarin 7‐glucoside (fermented broth); N: fraxidin (fermented broth); O: Aesculetin (fermented broth); P: fraxidin (mycelium). Q: (Z)‐6‐(2‐Methoxyvinyl)‐7‐methyl‐2H‐1‐benzopyran‐2‐one (mycelium); (F) The contents of unclassified coumarins in different periods. R: 3‐Hydroxycoumarin (fermented broth); S: dihydrocoumarin (fermented broth); T: liqcoumarin (fermented broth); U: 7‐Ethoxy‐4‐methylcoumarin (fermented broth); V: lternariol (mycelium); W: dihydrocoumarin(mycelium); X: 8‐hydroxy‐7‐methoxy‐3‐(2‐methylbut‐3‐en‐2‐yl)‐2H‐chromen‐2‐one(mycelium); (G) The intracellular and extracellular content of miltirone. (H) Heat map clustering of ABC transporters‐related differential compounds in fermentation broth. (I) Heat map clustering of ABC transporters‐related differential compounds in the mycelium.

To compare metabolites between the intra‐ and extracellular at different times, differential metabolites were screened out in both positive‐ion and negative‐ion modes. A total of 468 differential metabolites were found in the fermentation broth, with 255 having lower levels and 213 having higher levels (Tables S1 and S2). Similarly, 799 different compounds were found in the mycelium, with 494 having lower levels and 305 having higher levels, including lipids, organic acids and derivatives, flavonoids, steroids, alkaloids, terpenoids, etc. (Figure 6A,B; Tables S3 and S4). We also found different kinds of coumarins in *P. restrictum*. Ten of them were in the mycelium, and 14 were in the fermentation broth (Figure 6C–F). Remarkably, marmesin and aesculetin have been identified in *P. praeruptorum*, while other coumarins have not been reported. Marmesin is the most common coumarin and an important intermediate in the biosynthesis of coumarin. The content of marmesin was highest on day 4 of incubation. The content of terpenoids revealed an opposite trend in mycelium and fermentation, like the diterpene miltirone, suggesting their potential transmembrane transport in the fermentation process (Figure 6G). The analysis of differential compound enrichment showed that the ABC transporter enrichment map was highly enriched. The horizontal coordinates represent different experimental groups, the vertical coordinates represent the different metabolites compared in the group. Most of the differential compounds in the ABC transporter enrichment map were amino acids. The heat map analysis indicated that the extracellular content of all amino acids was decreasing except for L‐glutamine, which was first increasing and then decreasing. Except for the contents of L‐glutamic acid, L‐valine and L‐glutamine decreased first and then increased in cells. The contents of other amino acids increased first and then decreased (Figure 6H,I; Tables S5 and S6). The results showed that these differential metabolites were predominantly classified into coumarins, terpenoids and amino acids.

### KEGG pathway enrichment analysis

**FIGURE 7:**
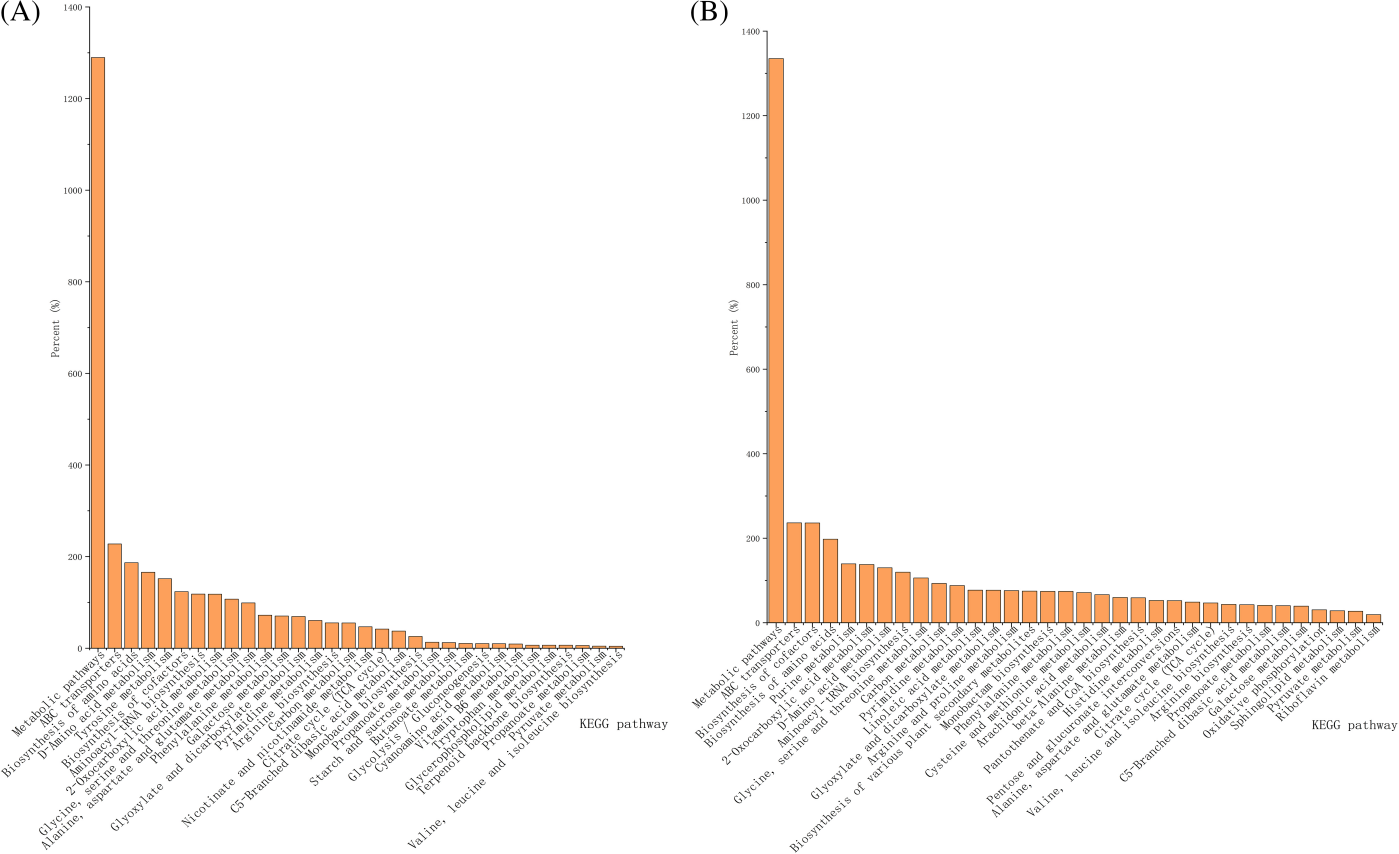
KEGG Classification of differential compounds in fermentation broth and mycelium. (A) KEGG Classification plot of fermentation broth; (B) KEGG Classification plot of mycelium.

The functional enrichment of the different compounds was further analysed over different periods (Figure 7). The results showed that the most significant intra‐ and extracellular enrichment was involved in metabolic pathways, and the terms with the highest degree of enrichment were biosynthesis of amino acids, ABC transporters, biosynthesis of cofactors, D‐amino acid metabolism and aminoacyl‐tRNA biosynthesis, phenylalanine metabolism, arginine biosynthesis, glyoxylate and dicarboxylate metabolism, alanine, aspartate and glutamate metabolism, riboflavin metabolism and citrate cycle (Tables S7 and S8). The bubble map of the metabolic pathways showed that the highly enriched extracellular pathways were alanine, aspartate and glutamate metabolism, glyoxylate and dicarboxylate metabolism, and terpenoid backbone biosynthesis (Figure 7A). Intracellular metabolic pathways were glyoxylate and dicarboxylate metabolism, alanine, aspartate and glutamate metabolism, glycine, serine and threonine metabolism, and purine metabolism (Figures S1 and S2; Tables S9 and S10). The classification of these metabolic pathways showed that these pathways were mainly classified into global and overview maps, amino acid metabolism, translation, and membrane transport. Amino acid metabolism, global and overview maps, and primary metabolism are closely related to life‐sustaining activities (Figure 7B). Phenylalanine (Phe) can undergo various chemical reactions to produce phenylpropane analogues, which in turn give rise to coumarins, compounds containing the coumarin structure. In addition, coumarin transport is dependent on ABC transporter proteins. Although there are significant differences in intracellular nucleotide metabolism, it was speculated that they might be related to the biosynthesis of intracellular ribonucleotides. Significant differences in the extracellular metabolism of terpenoids and polyketides were speculated to be related to extracellular terpenoid metabolism (Table 1).

**TABLE 1:** Classification of KEGG pathway enriched in intracellular and extracellular.

Location	Type	Pathways
Intracellular and intracellular enrichment	Amino acid metabolism	Alanine, aspartate and glutamate metabolism
		Glycine, serine and threonine metabolism
		Phenylalanine metabolism
	Carbohydrate metabolism	Glyoxylate and dicarboxylate metabolism
	Membrane transport	ABC transporters
	Translation	Aminoacyl‐tRNA biosynthesis
	Global and overview maps	2‐Oxocarboxylic acid metabolism
		Biosynthesis of amino acids
		Biosynthesis of cofactors
Extracellular‐specific enrichment	Metabolism of terpenoids and polyketides	Terpenoid backbone biosynthesis
Intracellular specific enrichment	Nucleotide metabolism	Purine metabolism

*Note*: Metabolic pathways with higher enrichment were screened by enriching differential compounds with VIP >1.25, *p* < 0.01.

The differential abundance score analysis was performed to determine the overall variation of all differential metabolites enriched in the same pathway (Figures S3 and S4). The DA analysis chart showed that terpenoid backbone biosynthesis showed a significant increase in the amount of compounds synthesized on day 2 compared to the other periods. On the 4th and 12th days of culture, the content decreased compared with other periods. It may be related to the excessive accumulation of extracellular secondary metabolites. The content of intracellular compounds on days 6 and 10 increased significantly compared with other periods, and the synthesis on days 2 and 12 decreased. Phenylalanine metabolism was down‐regulated in the fermentation broth on days 4 and 6. ABC transporters were downregulated throughout the whole period. Most of the compounds were highly expressed on day 1 compared to day 12 in the intracellular and extracellular compartments (Figures S5 and S6).

### *The deduced pathway of coumarin components in* P. restrictum

**FIGURE 8:**
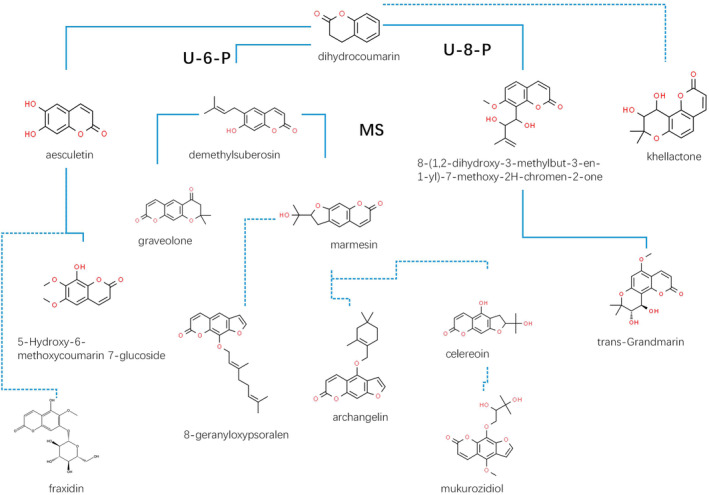
Biosynthetic pathways of coumarin in *P. restrictum* based on biogenic speculation. Structural formulae and names of compounds are indicated, arrows indicate the flow of metabolic reactions, and dashed lines indicate the flow of possible metabolic reactions.U‐6‐P (Umbelliferone 6‐propyltransferase); U‐8‐P (Umbelliferone 8‐propyltransferase); MS (Marmesin synthase).

**FIGURE 9:**
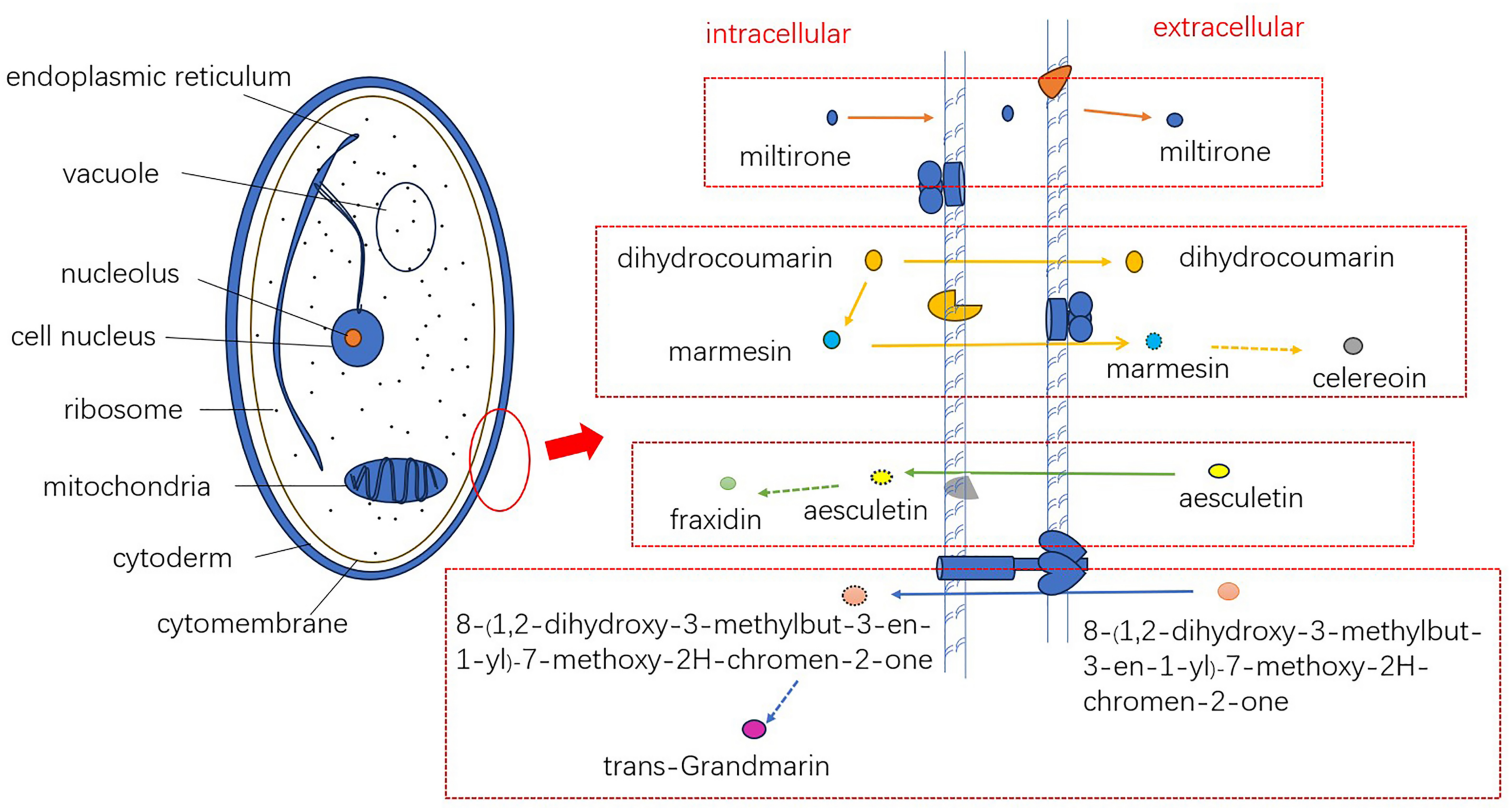
The working mode of transportation of the main metabolites in the intracellular and extracellular. The diagram on the left shows the structure of a fungal cell, and the diagram on the right shows the cell membrane and some of the terpenoids and coumarins presumed to be transported in the dashed box.

Metabolic pathways were tentatively predicted by the structural formulae and contents of all coumarins (Figure 8). The results showed that dihydrocoumarin contained demethylsuberosin, aesculetin, and 8‐(1,2‐dihydroxy‐3‐methylbut‐3‐en‐1‐yl)‐7‐methoxy‐2H‐chromen‐2‐one under the catalysis of umbelliferone 6‐prenyltransferase (U‐6‐P) and umbelliferone 8‐prenyltransferase (U‐8‐P), respectively, which further produced pyranocoumarin and khellactone. Through several steps of enzyme reactions, demethylsuberosin created the pyranosylcoumarins draveolone and marmesin. These compounds were then used to synthesize 8‐geranyloxypsoralen and archangelin, both of which belong to the furanosylcoumarin. The furanosylcoumarin celereoin, which can form mukurozidiol, a new antibiotic, is biologically related to marmesin. The 8‐(1,2‐dihydroxy‐3‐methylbut‐3‐en‐1‐yl)‐7‐methoxy‐2H‐chromen‐2‐one was the precursor to produce the pyranocoumarin trans‐grandmarin. Aesculetin is considered the precursor to the simple coumarins 5‐hydroxy‐6‐methoxycoumarin‐7‐glucoside and fraxidin. With the alterations in intracellular and extracellular coumarins, the conversion between intracellular and extracellular compounds will provide possible routes for coumarin transport (Figure 9). The results showed the cell membrane and some of the coumarins were presumed to be transported in the dashed box. The coumarin analogue dihydrocoumarin is translocated to the extracellular compartments while generating the intermediate product marmesin in the intracellular compartments, which may be retranslocated to the extracellular compartments to generate the product celereoin; esculetin may be translocated to the intracellular compartments to generate the product fraxidin; and 8‐(1,2‐dihydroxy‐3‐methylbut‐3‐en‐1‐yl)‐7‐methoxy‐2H‐chromen‐2‐one may be translocated intracellularly to produce trans‐grandmarin.

## DISCUSSION

### Variation of main differential metabolites in mycelia and fermentation broths

The individual size of *P. restrictum* gradually increases during the fermentation process. The mycelium gradually lengthens and becomes entangled with each other to form a ball by shaker culture. Meanwhile, the fresh weight and dry weight of mycelium also gradually increased as individual size increased. In addition, mycelium also produces a large number of secondary metabolites during the growth process. Previous studies have isolated the genera *Chaetomium*, *Aspergillus*, *Alternaria*, *Penicillium* and *Rotobacter* from the endophytes of Ginkgo. Some endophytes can produce large amounts of phytochemical defence substances, such as flavonoids, terpenoids and other compounds (Yuan et al., 2019). The intracellulars of the differential metabolites detected were mainly involved in amino acids and their derivatives, fatty acids, coumarins, glycerophospholipids, flavonoids, steroids, alkaloids and terpenoids. In addition, some secondary metabolic pathways are also the focus of our research. The metabolic pathway ABC transporters, which were highly enriched in differential compounds, were associated with coumarin synthesis, and similarly, terpenoid backbone biosynthesis is highly enriched in differential compounds, and terpenoids are also one of the important active components in Apiaceae.

### Changes in primary metabolites

Fungi have a crucial role in life processes by engaging in primary metabolism, which supplies plants with energy and building blocks for the synthesis of numerous essential chemicals. Among them, distinct amino acid metabolic pathways constitute integral parts of the plant immune system (Zeier, 2013). Amino acids related to phenylalanine, tyrosine, tryptophan, lysine and asparagine are important amino acids for plant resistance to pathogen attack. Some amino acid metabolic pathways are an important part of the plant immune system (Killiny & Hijaz, 2016). Lysine can induce pipecolic acid production during the production of salicylic acid, which also plays an important role in plant growth as well as parts of nitrogen metabolism in plants. The produced salicylic acid is mainly involved in the regulation of systemic acquired resistance (Yang & Ludewig, 2014). Tyrosine plays a key role in the synthesis of precursors for many secondary metabolites (Schenck & Maeda, 2018). The content of phenolic compounds was strongly influenced by pathogenic organisms. Plants treated with fungi contained high levels of phenolic compounds and higher resistance (Slatnar et al., 2016). Leucine is an important organic acid that plays a role in plant development and defence (Yuan et al., 2018). Phenylalanine metabolism is a central pathway in aromatic metabolism and an important precursor for a wide range of bioactive substances (Cai et al., 2016). Our results indicated that these amino acids are the precursors for the synthesis of several important secondary metabolites.

### Changes in secondary metabolites

C1 units are methyl donors and are associated with the biosynthesis of choline, purines, pyrimidines, etc. (Dartois et al., 2003; Yadav & Sundd, 2018). α‐Linolenic acid is involved in plant defence responses by activating JA‐mediated pathways (Dhakarey et al., 2016). Terpenoids are one of the most diverse and abundant classes of secondary metabolites among natural products, with tremendous structural and functional diversity and a range of important pharmacological and biological activities (Zhang et al., 2023). The biosynthesis of the terpenoid skeleton is responsible for the later formation of different terpenoids (Sun & Li, 2018). *Bupleurum chinense* promotes the synthesis of saikosaponin by affecting the terpene skeleton and triterpenoid biosynthesis (Yang et al., 2022). More investigation is needed to determine whether *P. praeruptorum* produces terpenes via rhizosphere fungi, thereby facilitating the production of saponins.

Iron deficiency is a factor that strongly induces coumarin secretion. Numerous studies have shown that ABC transporter proteins can mediate coumarin transport under iron deficiency conditions. AtABCG37 is involved in the secretion of highly oxygenated coumarins rather than the secretion of monohydroxylated coumarins. Coumarins from the cortex to the rhizosphere are dependent on the PDR9 transporter (Ziegler et al., 2017). Iron deficiency in *Nicotiana tabacum* induces coumarin secretion through the transport of *O*‐methylated coumarins to the rhizosphere, mediated by NtPDR3/NtABCG3 (Lefèvre et al., 2018). Esculin is a fluorescent coumarin glucoside that can be recognized by AtSUC2, and esculin is only translocated into the phloem to translocate to sink tissues via the AtSUC2 symporter (Robe et al., 2020). One of the MATE transporters, DTX18, is a transporter required for the translocation of hydroxycinnamic acid amides (HCAAs) to the leaf (Dobritzsch et al., 2016). Both MRP3 and MRP4 of multidrug resistance‐associated proteins (MRPs) belong to the ATP‐binding cassette family of efflux transporters. In addition, coumarin is deglycosylated prior to secretion, but the enzymes involved in its glycosylation and deglycosylation remain unresolved (Knox et al., 2018). Moreover, most of the current studies on coumarin transport focus on plants and animals in vivo, while microorganisms are rarely involved (Wittgen et al., 2012). Their subcellular localization and intracellular and extracellular transport are still poorly understood and deserve in‐depth studies. More interestingly, coumarin is an important intermediate in the biosynthesis of coumarin. Was this accumulation of mega‐high levels related to its vigorous synthesis and insufficiently timely conversion? Marmesin is a biologically essential precursor to furanocoumarins. The superiority of this bacterium is evident when several important enzymes for the synthesis of furanocoumarins in *E. coli* fail to show activity.

### *Possible transmembrane transport mechanisms of differential metabolites in* P. restrictum

ABC transporters function as individual active transporters that move substrates across biological membranes using ATP as an energy source (Bilsing et al., 2023). These efflux pumps carry a wide range of compounds across biological membranes, including phospholipids, peptides, steroids, polysaccharide amino acids, organic anions, bile acids, drugs and other exogenous substances. Our results indicated that the ABCB and ABCG subfamilies are involved in the specific synthesis of small molecules in mycelium. The ABCB subfamily may be involved in the extracellular transport of some growth factors. ABCB1 is an identified growth hormone efflux carrier in many higher plants (Liu et al., 2017; Ma & Han, 2016; Yang & Murphy, 2009). ABCB10 is situated within the inner mitochondrial membrane and can transport a crucial molecule that plays a vital role in avoiding oxidative damage (Cao et al., 2023). Abscisic acid leaves the root xylem and comes into the leaf stomatal cells through ABCG transporters. This can lower the amount of water that is lost through transpiration. Also, ABCG transporters are very important for getting rid of important hormones that cause biological stress, like jasmonic acid and salicylic acid, along with other substances that are produced naturally. In this way, they protect plants as a first line of defence against pathogen damage (Dhara & Raichaudhuri, 2021). ABC transporter proteins and their subfamilies are involved in a variety of processes within the mycelium, such as pathogen response, diffusion barrier formation or phytohormone transport, and have an impact not only on the microorganisms themselves but also on the symbiotic plants (Song et al., 2023).

## CONCLUSION

Throughout the growth and maturation of *P. restrictum*, the mycelium intertwines and forms clumps, while concurrently producing a large amount of secondary metabolites. There are evident changes in the amount and types of metabolites during different fermentation periods, and intracellular and extracellular metabolism are in a state of perpetual flux and interchange. It is thought that the strain backing happened on the fourth day since there was the highest concentration of marmesin, which is a typical precursor of furanocoumarin. This may be an important point for the root growth of *P. praeruptorum*. Moreover, our results demonstrated that the mycelium was able to produce novel coumarins that were not observed in *P. praeruptorum* during fermentation, which is a better addition both as a discovery of new coumarins and as a route for heterologous production. The reason for the effects of *P. restrictum* and its secondary metabolites on the early bolting of *P. praeruptorum* is still unclear when they are inoculated. The metabolomics analysis of the rhizosphere microbiome will provide a scientific reference to solve the problem of the effect of rhizosphere microbial backing on the plant and its interactions.

## AUTHOR CONTRIBUTIONS

**Yuanyuan Wang:** Data curation (equal); formal analysis (equal); investigation (equal); methodology (equal); software (equal); validation (equal); visualization (equal); writing – original draft (equal); writing – review and editing (equal). **Ranran Liao:** Formal analysis (equal); investigation (equal); methodology (equal); validation (equal); writing – original draft (equal). **Haoyu Pan:** Formal analysis (equal); investigation (equal); methodology (equal); writing – original draft (equal). **Xuejun Wang:** Formal analysis (equal); resources (equal); supervision (equal); writing – review and editing (equal). **Xiaoting Wan:** Formal analysis (equal); investigation (equal); methodology (equal); writing – original draft (equal). **Bangxing Han:** Conceptualization (equal); funding acquisition (equal); project administration (equal); supervision (equal); writing – review and editing (equal). **Cheng Song:** Conceptualization (equal); data curation (equal); funding acquisition (equal); methodology (equal); project administration (equal); resources (equal); supervision (equal); writing – original draft (equal); writing – review and editing (equal).

## CONFLICT OF INTEREST STATEMENT

The authors declare no conflict of interest.

## Supporting information


**Figure S1.** Bubble diagram for metabolic pathway analysis of differential metabolites in fermentation broth at different periods.

**Figure S2.** Bubble diagram for metabolic pathway analysis of differential metabolites in mycelium at different times.

**Figure S3.** DA Score plots of differential KEGG metabolic pathways in fermentation broths from different periods.

**Figure S4.** DA score plots of differential KEGG metabolic pathways in mycelium at different periods.

**Figure S5.** Diagram of the KEGG Pathway of ABC transporters in fermentation broth. The dots in the figure indicate metabolites, with bright red representing up‐regulated significantly different metabolites and bright blue representing down‐regulated significantly different metabolites; boxes indicate genes (proteins) involved in the pathway, and green boxes indicate validated genes (proteins) involved in the pathway; and the connecting lines indicate the direction of flow of the metabolic reactions.

**Figure S6.** Diagram of the KEGG Pathway of ABC transporters in mycelium. The dot in the figure indicates metabolites, with bright red representing up‐regulated significantly different metabolites and bright blue representing down‐regulated significantly different metabolites; boxes indicate genes (proteins) involved in the pathway, and green boxes indicate validated genes (proteins) involved in the pathway; and the connecting lines indicate the direction of flow of the metabolic reactions.

**Table S1.** Differential compound screening‐fermented broth.

**Table S2.** Differential compound screening‐mycelium.

**Table S3.** The pie data matrix of the fermentation broth.

**Table S4.** The pie data matrix of the mycelium.

**Table S5.** Differential compounds of fermentation broth.

**Table S6.** Differential compounds of mycelium.

**Table S7.** KEGG pathway classification of fermentation broth.

**Table S8.** KEGG pathway classification of mycelium.

**Table S9.** Pathway analysis of fermentation broth.

**Table S10.** KEGG enrichment analysis of metabolic pathways.

## Data Availability

The raw data generated and analysed during the study are available at figshare: https://doi.org/10.6084/m9.figshare.24471793.v1.
