# Supplementary figures and images for: Comparative metabolic profiling of the mycelium and fermentation broth of Penicillium restrictum from Peucedanum praeruptorum rhizosphere
Source: Environ Microbiol Rep. 2024 Jun 6;16(3):e13286. doi: 10.1111/1758-2229.13286 (PMC11156492; doi:10.1111/1758-2229.13286)

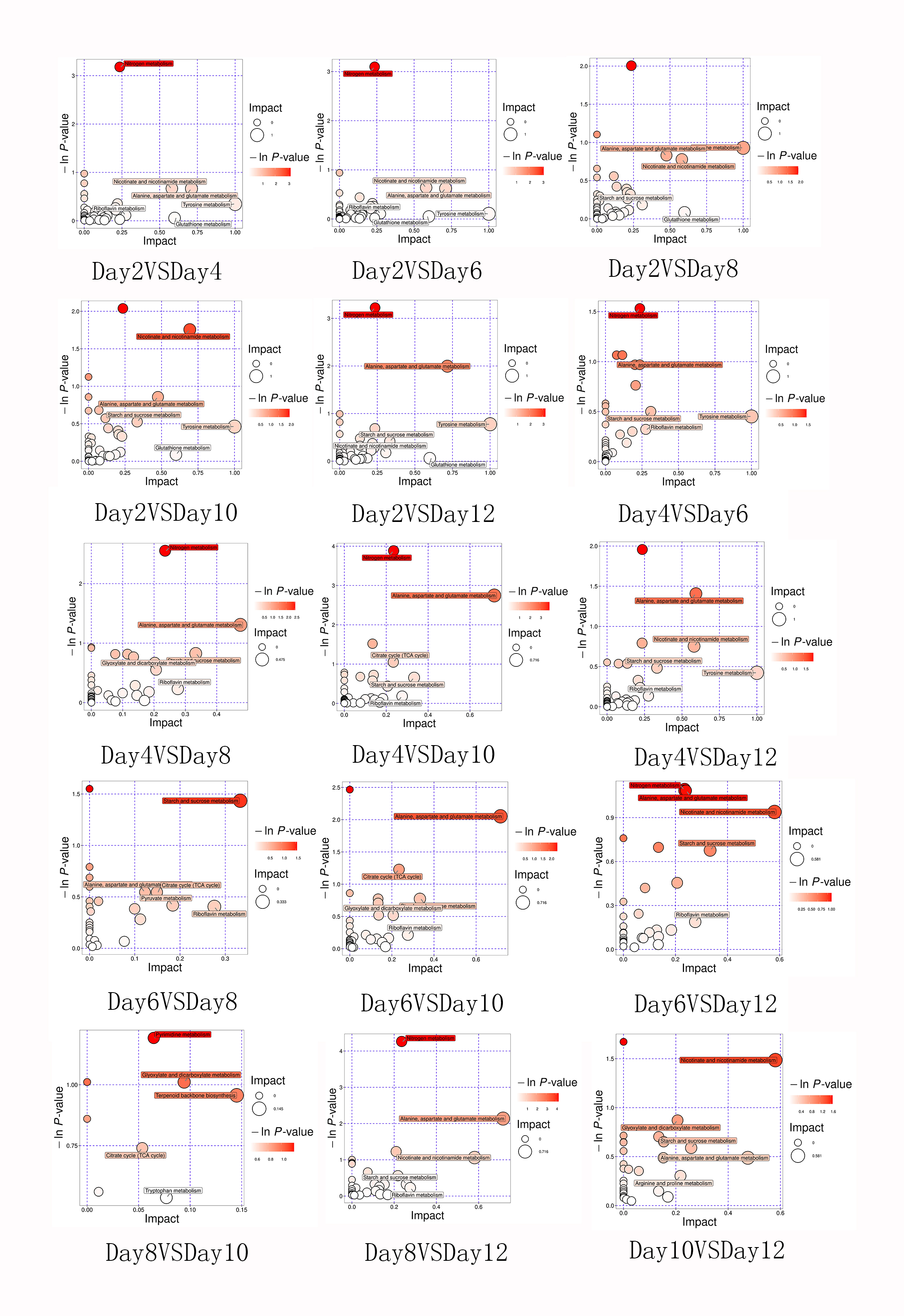

Supplement: Supplementary file 1 — Figure S1. Bubble diagram for metabolic pathway analysis of differential metabolites in fermentation broth at different periods. [file EMI4-16-e13286-s001.jpg]

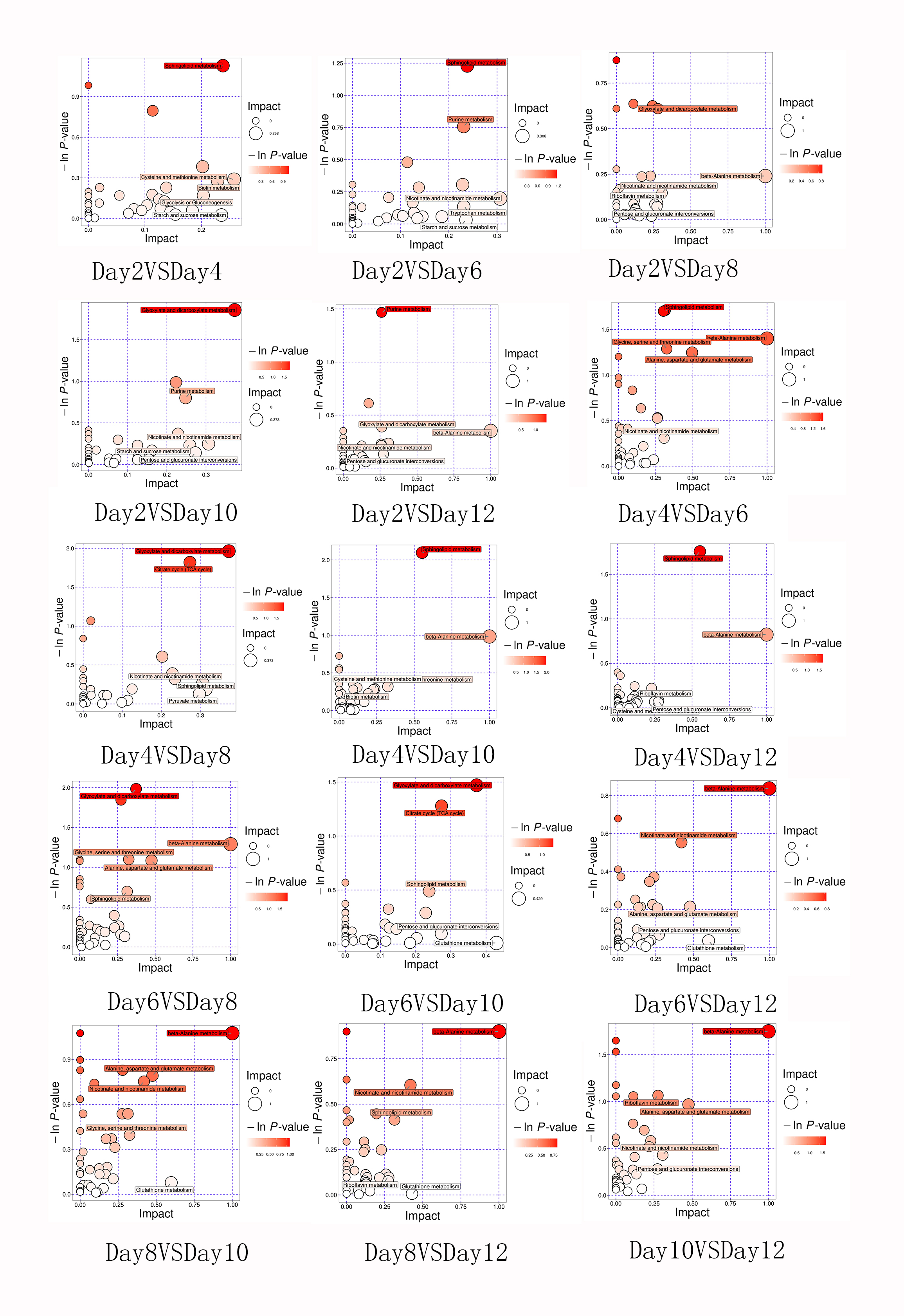

Supplement: Supplementary file 2 — Figure S2. Bubble diagram for metabolic pathway analysis of differential metabolites in mycelium at different times. [file EMI4-16-e13286-s016.jpg]

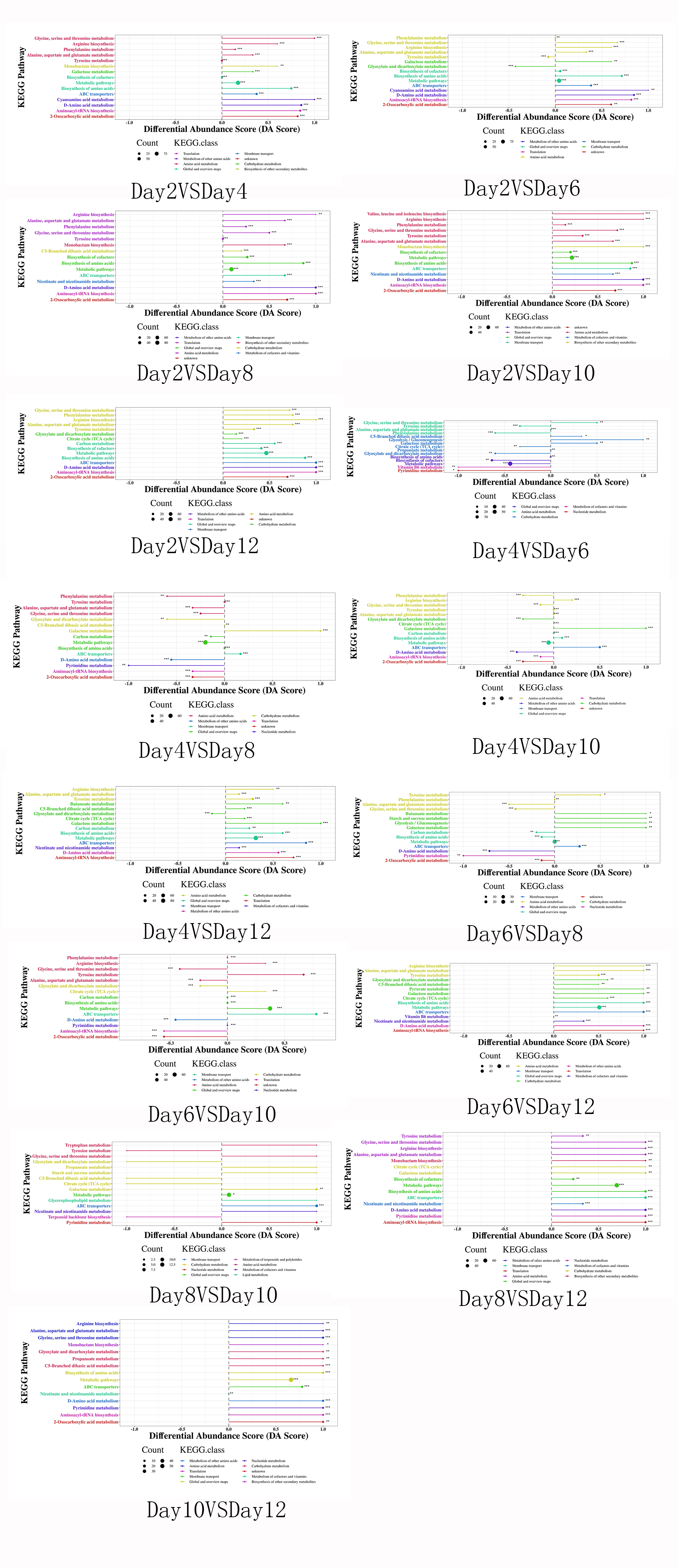

Supplement: Supplementary file 3 — Figure S3. DA Score plots of differential KEGG metabolic pathways in fermentation broths from different periods. [file EMI4-16-e13286-s006.jpg]

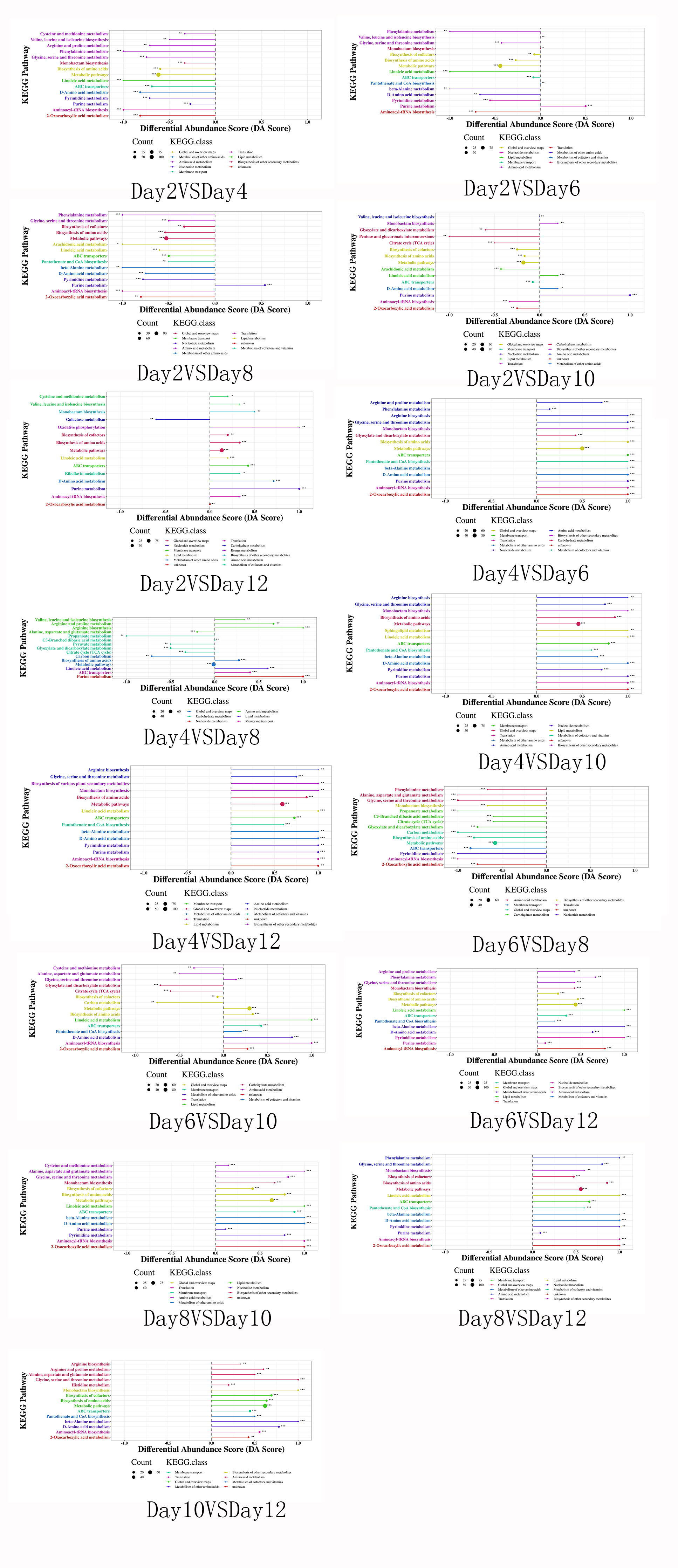

Supplement: Supplementary file 4 — Figure S4. DA score plots of differential KEGG metabolic pathways in mycelium at different periods. [file EMI4-16-e13286-s002.jpg]

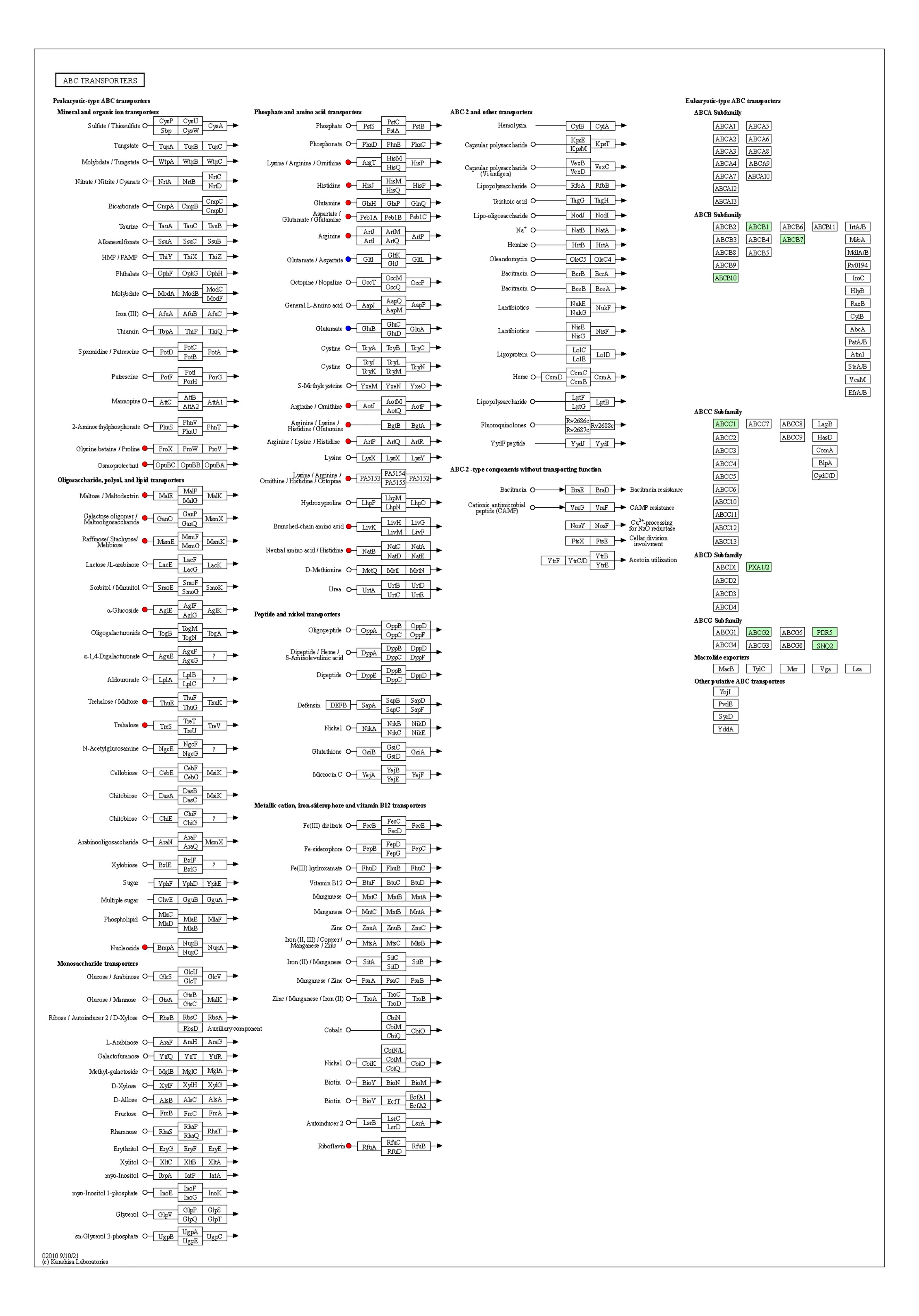

Supplement: Supplementary file 5 — Figure S5. Diagram of the KEGG Pathway of ABC transporters in fermentation broth. The dots in the figure indicate metabolites, with bright red representing up‐regulated significantly different metabolites and bright blue representing down‐regulated significantly different metabolites; boxes indicate genes (proteins) involved in the pathway, and green boxes indicate validated genes (proteins) involved in the pathway; and the connecting lines indicate the direction of flow of the metabolic reactions. [file EMI4-16-e13286-s013.jpg]

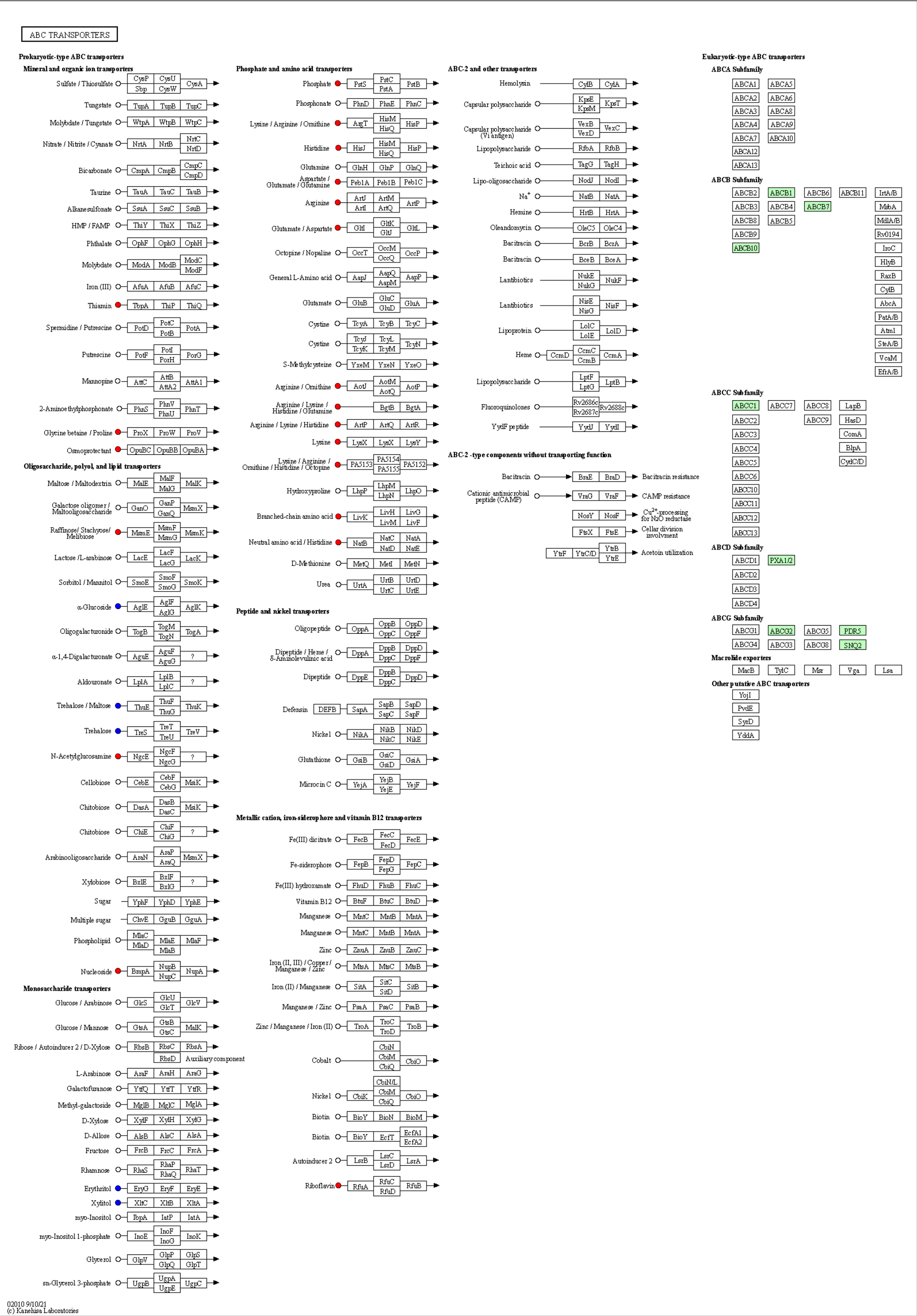

Supplement: Supplementary file 6 — Figure S6. Diagram of the KEGG Pathway of ABC transporters in mycelium. The dot in the figure indicates metabolites, with bright red representing up‐regulated significantly different metabolites and bright blue representing down‐regulated significantly different metabolites; boxes indicate genes (proteins) involved in the pathway, and green boxes indicate validated genes (proteins) involved in the pathway; and the connecting lines indicate the direction of flow of the metabolic reactions. [file EMI4-16-e13286-s012.jpg]
